# A data-driven framing of player and team performance in U.S. Women's soccer

**DOI:** 10.3389/fspor.2023.1125528

**Published:** 2023-03-08

**Authors:** Sachin Narayanan, N. David Pifer

**Affiliations:** Department of Sport Management, Florida State University, College of Education, Tallahassee, United States

**Keywords:** gender, competitive advantage, performance analytics, football (soccer), america

## Abstract

**Introduction:**

In establishing historical benchmarks for success on the pitch and striving to achieve parity off it, the United States Women's National Team (USWNT) and the National Women's Soccer League (NWSL) have long served as standard bearers for professional women's soccer around the globe. However, off-field dilemmas and incessant juxtapositions to men's soccer frequently overshadow the elements that make U.S. women's soccer unique; that is, in the quest to expose and rid the women's game of blatant misconduct, discriminatory practices, and negative stereotypes, relatively little attention has been devoted to performance features that separate the U.S. women's soccer product from its competition. Because many of the issues hindering the progress of women's soccer are rooted in media and managerial practices that marginalize or ignore its positive traits, a need exists for analyses that will properly identify its innate characteristics and competitive advantages so that media members, managers, and fans can accurately frame their perceptions of women competing in the sport.

**Methods:**

To this end, we collected reliable samples of public event data from 560 professional soccer matches and used ANOVAs and t-tests to identify the characteristics that distinguish U.S. women's soccer from other professional leagues and teams.

**Results and Discussion:**

In doing so, we showed that the USWNT tends to shoot from more opportune areas and press opponents at a higher rate, and that the NWSL has recently been matched in quality across certain performance metrics by England's FA Women's Super League.

## Introduction

Professional women's soccer has seen its popularity rise in recent years, with both club and international competitions setting new marks for viewership and attendance. The 52 matches played in the 2019 FIFA Women's World Cup, for example, were viewed by over 1.12 billion people worldwide, and the final between the United States and Netherlands held an average live audience of 82.18 million that made it the most watched FIFA Women's World Cup match in global broadcast history. More recently, a 2022 UEFA Women's Champions League quarterfinal between Barcelona and Real Madrid saw the former claim a 3–1 home win over its Spanish rival in front of 91,553 spectators (1). This set a new world record for attendance at a women's soccer match, eclipsing the prior mark of 90,185 set at the 1999 FIFA Women's World Cup Final between China and the United States.

For those well-versed in women's sport, it is not surprising to find the United States Women's National Team (USWNT) at or near the top of many of these achievements. The USWNT has won four of the eight (50%) FIFA Women's World Cups that have been played since 1991 (2) and has never finished worse than third (3). It also boasts four Olympic gold medals amid support from youth and professional club systems that have helped maintain its elite status. Historically, the National Women's Soccer League (NWSL)—the United States' premier women's club league—has held strong ties to the USWNT, with players contracted to both earning higher salaries and extra attention from sponsors and supporters. The combined efforts of the USWNT and NWSL have allowed U.S. women's soccer to serve as a prototype for professional women's soccer, providing models for attracting and retaining talent at the club level and achieving success in international competitions.

However, U.S. women's soccer, like many of its compatriots in professional women's sport, is often marginalized against the backdrop of men's sport and a society that tends to filter its understanding of athletic skill through a male orientation. Female athletes and competitions, in comparison to the men, must contend with limited publicity and sexualized stereotypes that focus more on features of physical attractiveness than athletic prowess. The elements that make them unique from a performance perspective are frequently ignored or perceived negatively in relation to the standards established by their male counterparts. Thus, rather than highlighting and promoting the elements that positively distinguish female sports, media members, analysts, and consumers fixate on their perceived shortcomings relative to the men.

Perhaps no better illustration of this is needed than the debate that ensued during a panel discussion at the 2020 Laureus Sports Awards in Berlin. Fabio Capello, a former professional soccer player in Italy and manager of the England men's team, was asked what women's soccer needed to do, on the whole, to improve on a successful 2019 World Cup. “I think the goal is too big for women and that the pitch is too wide,” said Capello. “When they [women] play basketball and volleyball they lower the net because they are not tall like men. I think the size of the goals makes it really difficult for the keeper, because in football, you have to jump,” (4). His response suggested that goalkeeping standards were still not at a level sufficient to the men's game, and that the rules and regulations of the game should be altered to accommodate these perceived shortcomings. Similar assertions have been made in other sports, like basketball, often without consideration of the underlying implications or sexist undertones (5).

Ultimately, if female sports are to be viewed as more than subsidiaries to the men's versions of their games, more work needs to be done to highlight the elements that make them unique. This is particularly true in relation to athletic performance, an area of sport that has become more accessible in the modern era of big data and analytics. Prior research, owing much to the limited availability of detailed performance data, has done little to identify the playing skills and abilities common to successful female sports entities like U.S. women's soccer. While physiological differences between female and male athletes have been analyzed at length, technical differences between men's and women's teams have been explored to a lesser extent. Given the opportunity for data-driven insights to help reframe negative stereotypes and develop schemas more appropriate to women's sports, researchers would be wise to undertake such efforts while focusing on teams that have served as standard bearers for success.

Therefore, the purpose of this study was to explore and identify the distinct performance features that have made U.S. women's soccer unique on the pitch. In doing so, we provide a better understanding of the characteristics and competitive advantages that have allowed U.S. women's soccer to develop into one of the most successful entities in professional sport, further facilitating the development of frameworks that are more suited to the women's game. With various European countries such as England, France, and Germany now starting to adopt the American model and invest extensively in their women's soccer programs (6), this study further serves as a guide to administrators and decision-makers looking to identify the areas that have helped distinguish the USWNT and NWSL from its competitors. To accomplish these ends, we use public, detailed event data from StatsBomb, comparative statistical analyses, and insightful data visualizations to answer the following research questions:

- RQ1. How do the key performance metrics of the USWNT differ from those of other international women's soccer teams?
- RQ2. How do the key performance metrics of the USWNT differ from international men's teams across the world?
- RQ3. How do the key performance metrics of clubs in the National Women's Soccer League (NWSL), differ from those of England's FA Women's Super League (WSL)?

In exploring these differences, we are able to outline the tactical and technical state of women's soccer in North America and analytically explore its first-mover advantages, both in comparison to its direct competitors in the women's game and the men's competitions that have traditionally shaped perceptions of the sport.

## Literature review

### The evolution of U.S. Women's soccer

Women's soccer first appeared at the club level in England during the late 1800s, and by the 1920s record-breaking attendances of over 53,000 were already becoming the norm (7). However, the sport's early efforts to grow were curtailed by the English Football Association (FA) in the form of a ban from using the FA's pitches. The extremely physical nature of the game back then, in addition to the apparently higher possibility of financial fraud, were given as excuses by the English FA for the ban (8). Once the ban was lifted in the 1970s, most of the European countries followed suit and numerous women's soccer associations were created.

While the early 1970s and 1980s played host to preliminary versions of an international tournament, it was not until 1991 that the Women's World Cup was officially approved by FIFA. Taking place in China, the tournament saw the United States lift the trophy after defeating Norway 2–1 in the final. Although the United States did not have a great history in the men's tournament, winning the first international women's soccer title proved to be a major boost for growth of the sport in the country. The subsequent World Cups provided numerous moments for fans to cherish, with some games clocking record attendances of over 90,000 (9). More recently, over 1.12 billion fans around the world viewed the official broadcast of the 2019 FIFA Women's World Cup in France, with the final game at one point peaking at 263.62 million unique viewers. This happened on the same day that the Gold Cup and Copa America men's finals were played, indicating how much the sport of women's soccer had grown (10).

The introduction of the Title IX Omnibus Education Act of 1972 in the United States, which happened to coincide with the removal of the playing bans for women's soccer in Europe, brought forth a new era of female sport participation in the United States. By prohibiting sexual discrimination at the collegiate level and requiring schools to devote more resources toward equal opportunities for women in sport, Title IX encouraged more investment in women's athletic programs like soccer (11). The lack of a dominant men's soccer league in the U.S. during this time was also a contributing factor, as it ensured some of the initial supporters could arrive without rigid, preconceived notions of soccer being a masculine sport. Following tremendous development at the grassroots level, women's soccer quickly developed into a reputable sport on the domestic and global fronts. By winning the inaugural FIFA Women's World Cup in 1991 and following that success with victories at the 1996 Olympic Games and 1999 FIFA Women's World Cup, the USWNT quickly established itself as a global soccer force (12).

A stable professional women's club league arrived in the U.S. in 2012 when the National Women's Soccer League (NWSL) was established to fill the void left by the dissolved Women's United Soccer Association (WUSA) and Women's Professional Soccer (WPS) leagues (13). While initially focusing on talent from the North American continent, the NWSL gradually grew into a landing spot for some of the sport's global stars. Even so, the NWSL managed to maintain its connections to top domestic talent through a robust draft process and collective bargaining agreements that encourage the top USWNT players to remain in the NWSL (14).

### Perceptions of Women's soccer and female sport

Framing theory suggests that the way in which something is presented to an audience (e.g., sport being communicated to viewers through the media) will influence the resulting perceptions of the object being framed (15). To this end, women's soccer, like many other women's sports, has faced numerous challenges in its quest to gain distinction amidst the stereotypes, biases, and systemic blockades that have been formed by misaligned framings. A lack of quantity and quality in media coverage, for instance, has frequently led to perceptions of women's sports as second-rate offerings. Lebel & Danylchuk (16) suggest that the marginalized and comparatively disproportionate amount of coverage devoted to women's sports by the media limits awareness and causes the public to equate a lack of coverage with a lack of importance (16). In some cases, the media goes so far as to directly question the basic competencies of female players, coaches, and officials on the basis of their sex. For example, when a female referee came on as a substitute for a male referee in a 2010 men's game between Coventry City and Nottingham Forest, the local newspapers went with the headline “Can Women Referee Men's Footie,” and “Oi, Ref! Are You Blonde?” (15, p. 331). In addition, the aforementioned assertions of Capello reflected a perceived inability for females to perform adequately in goal (3). Despite fans in many sports favoring more offense (scoring), he felt the product was soured by blatant errors and physical differences on the goalkeeping front.

In other instances, women's sports are oversexualized or framed in a manner consistent with society's preconceived notions of femininity. The media has traditionally categorized sports into masculine (football, soccer, basketball, and hockey) and feminine (individual sports such as gymnastics and figure skating) categories based on the traditional expectations of male and female athletes, creating stereotypes that are hard to break (17). When female athletes receive media coverage, emphasis is often placed on bodily features and traits of physical attractiveness that are based outside of sport (18). This trend was evident during the heightened success of the USWNT in 1999 when the nature of Brandi Chastain's celebration was scrutinized to a greater extent than her game-winning exploits on the pitch. After scoring the winning penalty in the top left corner with her weaker foot, Chastain became the subject of media attention because she removed her jersey as part of her celebration; rather than analyzing how she had just won her team the World Cup trophy with a remarkable piece of skill, media members focused on the celebration and used it as an opportunity to label her as a “controversial” figure (12).

In some cases, women's sports are simply positioned as lesser offshoots to the male versions of their games, lending to a form of gender-bland sexism that treats female sports and subjects as secondary to the men's sports that receive most of the attention (19). That men's sports receive most of the attention is explainable, in part, by their earlier establishment and the inner workings of the gender schema theory (20). Prior research has shown that the schemas — cognitive networks of associations that guide an individual's perceptions — activated by competitive sport contexts align with masculine features (21), causing society to associate sports with the male gender. In being one of the few team sports where both men and women play by an identical set of rules, women's soccer is at an elevated risk for being compared to the men's version. This makes the establishment of frameworks specific to women's soccer all the more important.

### The need for an analytical approach

The applicability of Goffman's (15) frame analysis and Bem's (20) gender schema theory indicate the presence of gender-based constructs in soccer. That is, there are paradigms rooted in the men's version of the sport that are used to frame mediated perceptions of women's soccer. When observing or analyzing the sport, this diverts attention away from the elements that make it unique and interesting, instead positioning the product as an inferior byproduct of the men's game. Thus, we need to take a closer look at what happens on the pitch during games in order to better quantify performance and isolate features that distinguish U.S. women's soccer.

In prior research, such approaches have generally been taken from physiological perspectives. Evolutionary psychologists have found evidence of increased competitiveness in men's sports due to longer, intense training, variations in the hormonal regulation of body fat, differences in cardiovascular and oxygen intake, and a higher susceptibility to injuries in women (22). Pedersen, Aksdal and Stalsberg (23) found that female soccer players have a lower muscle mass that requires them to exert more energy in movement, and that female goalkeepers suffer from a comparative lack of height. Additional research has shown that male athletes rank higher in such categories as endurance, kicking velocity, speed, strength, jumping, and stride length (24–26), suggesting that many of the perceived differences may be rooted in athletic, rather than skill-based, differences.

With the advent of open-source data in the last decade, researchers have started taking a more analytical approach to finding technical performance differences in women's and men's soccer. Some major differences that have been identified are that women's teams tend to score more goals and win games by larger margins at international tournaments (27). Though fans are generally understood to value offense and goal scoring (28), lopsided victories may contribute to fans' perceptions of women's soccer as less challenging or skillful (16). At the club level, (29) found that women tend to shoot from different locations than men and convert certain types of shots at a higher rate (Bransen and Davis, 2021). All else equal, female shot takers scored more frequently from close range and took fewer shots from the top of the 18-yard box.

Pappalardo, Rossi, Natilli and Cintia (30) used 2018 Men's World Cup and 2019 Women's World Cup match event data to analyze performance differences across gender. Findings revealed that the average women's match featured significantly less fouls, fewer passes, shorter and slower passes, lower pass accuracies, more shots from close range, and faster recovery times after the ball was lost. The women were also significantly quicker at resuming play following restarts. A study by Garnica-Capparós and Memmert (31) unearthed similar trends, finding that female players in international matches perform more passes but with less accuracy, win fewer ground duels, and clear the ball more frequently than men. Based on their findings, Pappalardo's study also labeled the women's game as being more “loyal” than the men's game in the sense that female players committed fewer fouls and took less time to resume their matches following stoppages in play. However, they also characterized women's games as being more “fragmented” due to teams exchanging possession of the ball more frequently and displaying less accuracy in their passes.

While these select papers provide insight on some key performance indicators in women's soccer, they largely ignore the theoretical connections and specific implications their findings hold across different segments of sport and society. Likewise, prior studies exploring gender schemas and other theoretical frameworks have rarely incorporated granular performance data in their assessments. Clément-Guillotin and Fontayne (32), for example, used survey data to identify a gender schema in competitive sport, but no attempts were made to define this schema in more specific contexts. In addition, most of the data in the comparative studies were from small samples and lower quality sources. With data quality and reliability considered to be highly important in soccer analytics (33), reputable data collection companies such as StatsBomb and Stats Perform (formerly known as Opta) should be used. StatsBomb, for instance, boasts one of the most extensive public archives of detailed soccer event data that it publicly shares to promote research in the field. Data from numerous women's soccer matches, including the USWNT, NWSL, and other high-level international and club competitions, are featured in this repository, providing our examination and others with a rare opportunity to conduct analyses on female sports data.

## Materials and methods

### Design

To distinguish U.S. women's soccer from competing versions of the sport, we collected samples of detailed event data from StatsBomb's open-source repository on GitHub. StatsBomb is a reputable data provider in the soccer industry that has partnerships with various professional clubs and leagues. The public data they provide affords researchers the following advantages: (a) they allow for analyses to be reproduced and replicated, confirming their validity; (b) they include rare samples of USWNT and NWSL data, as well as data from other leagues of interest, and (c) they are collected and processed through a multi-level, quality control process, helping ensure reliability. StatsBomb also has dedicated documentation, packages, and tools in multiple programming languages that allow for easy analysis of their data. A list of the various competitions and seasons that were examined in this study is provided in Table 1.

**Table 1:** Various players, teams, and competitions for which free data was available.

Women's Soccer	Men's Soccer
FA Women's Super League (2018–2021)	FIFA Men's World Cup (2018)
National Women's Super League (2018)	UEFA Men's Euros (2020)
FIFA Women's World Cup (2019)	
UEFA Women's Euros (2022)	

The collected data were representative of 1,491,626 women's soccer events from 1,081 different players (445 matches) and a total of 420,579 men's soccer events from 973 different players (115 matches). The data were filtered to select the key actions that were highlighted in prior literature (34, 35) as being indicative of player and team performance. With goals being the single most important performance metric in soccer, emphasis was placed on data related to shots and events that prevented or led to high quality chances. These included actions classified by StatsBomb as “Shot”, “Goal”, “Pass”, “Block”, “Clearance”, “Dribble” (an attempt by a player to take the ball past an opponent), “Duel” (winning a 50–50 battle against an opponent), “Interception”, “Miscontrol”, “Dispossessed”, and “Fouls Committed” (36). All data points had corresponding location coordinates and timestamps that helped identify where and when the action took place on the field. Information regarding player positions, the outcome of each event, a more detailed description of each event (e.g., type of shot, pass, tackle, or duel), and the type of play pattern the event was involved in were also assigned to each observation.

### Procedures

Following their collection, the events were split into five different datasets according to the various groups mentioned in the research questions. These were (a) the United States Women's National Team (USWNT), (b) the other women's national teams (OWNT), (c) the National Women's Super League (NWSL) clubs, (d) the English FA Women's Super League clubs (FAWSL) and (e) the men's national teams (MNT). All the values were recorded at the team level as “per-90” statistics for each team, meaning the stats were standardized to represent a team's average performance over the 90-min of a typical soccer match. Analyses of variance (ANOVAs) and independent sample t-tests were then conducted to identify significant differences between the U.S. women's teams and teams in the other groups across their 90-min averages for these stats. Intuitive data visualizations (e.g., heat maps and shot plots) related to some of the key metrics were also created to provide alternative views of the results.

### Statistics

ANOVAs are frequently used in situations where the means of several groups need to be compared to the mean of a reference group. Using an *F*-test, the means are compared for significant statistical differences, with *p*-values less than .05 indicating significantly different means. In situations where we are only comparing the means of two distinct groups, independent sample *t*-tests suffice. Given our aims to compare the mean performance metrics of U.S. women's soccer at the national and club levels (i.e., the reference groups) to the average metrics of the other groups (e.g., other women's national teams, other women's club teams, and men's national teams) ANOVAs and t-tests provided manageable and reliable methods for addressing the stated research questions. In conjunction with these statistical approaches and their associated hypothesis tests, descriptive statistics were further examined through detailed data visualizations.

## Results

Two sets of comparisons were constructed to address the research questions, one focusing on the international teams to highlight the differences between the USWNT and the groups of other national teams, and another identifying significant differences between the NWSL and the English FA Women's Super League (FAWSL). To answer RQ1, a one-way ANOVA for three groups (USWNT, OWNT and MNT) was utilized, with the USWNT serving as the reference group for comparison. For RQ2, an independent samples t-test was conducted on the averages of the two groups (NWSL and FAWSL).

### Differences between the USWNT and other national teams

Table 2 displays the results of the one-way ANOVA comparing the USWNT to other women's national teams (OWNT) and the men's national teams (MNT) in the data. From this, it was observed that the USWNT took a significantly higher number of shots than the other national teams, *F*(2,202) = 6.53, *p* < .01 (*M* = 18.57, *SD* = 10.91). Accordingly, they scored goals at a significantly higher rate than the other teams, *F*(2,202) = 11.00, *p* < .01, averaging 3.57 goals per 90 min in comparison to the tallies of 1.26 (OWNT) and 1.47 (MNT) averaged by the other groups. The USWNT appeared to apply much of its offensive pressure early, taking significantly more shots in the first half compared to the other groups, *F*(2,202) = 13.73, *p* < .01 (*M* = 9.71, *SD* = 4.15); however, the number of shots taken in the second half was not significantly different from the other groups, likely because they were already leading their opponents at this stage.

**Table 2:** ANOVA Comparing USWNT Stats (Per 90 Min) to Other National Teams

Team	USWNT		OWNT		MNT			
Number of Games	7		83		115			
	Mean	SD	Mean	SD	Mean	SD	*F*	*p*
Shots	18.57	10.91	12.41	4.21	13.02	3.79	6.53	0.00
Goals	3.57	4.19	1.26	0.84	1.47	1.16	11.00	0.00
Successful dribbles	11.57	5.38	7.56	2.9	6.33	2.39	14.93	0.00
Unsuccessful dribbles	15.14	8.57	10.56	3.97	9.26	3.24	9.44	0.00
Shots in 1st half	9.71	4.15	5.6	2.24	5.54	1.73	13.73	0.00
Shots in 2nd half	8.86	7.49	6.52	2.24	6.58	2.19	2.82	0.06
Goals in 1st half	1.57	1.13	0.56	0.62	0.44	0.46	13.68	0.00
Goals in 2nd half	2	3.61	0.65	0.5	0.77	0.61	8.43	0.00
Comp. Passes p90	389.14	144.67	331.24	78.69	417.19	75.29	28.14	0.00
In Comp. Passes p90	113.43	12.99	109.56	23.61	94.55	18.79	13.86	0.00
Blocks	1.33	0.58	1.05	0.55	1.18	0.73	1.27	0.28
Clearances	23.86	15.74	24.64	8.82	12.05	11.81	33.70	0.00
Interceptions	3.14	1.86	6.13	3.41	5.95	3.08	2.84	0.06
Duels	6.2	4.64	10.49	4.04	7.59	3.15	17.54	0.00
Miscontrols	10.71	3.49	15.11	4.1	12.21	3.07	18.20	0.00
Dispossessions	12.71	3.45	14.19	4.64	9.89	3.41	28.69	0.00
Fouls committed	3.33	2.58	10.34	3.71	14.22	3.47	51.90	0.00

Outside of shots and goals, the USWNT performed a higher number of dribbles, which led to significantly higher rates of successful dribbles, *F*(2,202) = 14.93, *p* < .01 (*M* = 11.57, *SD* = 5.38), and unsuccessful dribbles, *F*(2,202) = 9.44, *p* < .01 (*M* = 15.14, *SD* = 8.57). The USWNT also averaged significantly more completed passes, *F*(2,202) = 28.14, *p* < .01 (*M* = 389.14, *SD* = 144.67) and incomplete passes, *F*(2,202) = 13.68, *p* < .01, (*M* = 113.43, *SD* = 12.99), per 90 min compared to the OWNT group; however, the MNT group averaged significantly more successful passes (*M* = 417.19, *SD* = 75.29) and significantly fewer unsuccessful passes (*M* = 94.55, *SD* = 18.79) per 90 min compared to the USWNT.

Continuing, the USWNT had significantly fewer clearances, *F*(2,202) = 33.70, *p* < .01 (*M* = 23.86, *SD* = 15.74), and won significantly fewer duels, *F*(2,202) = 17.54, *p* < .01 (*M* = 6.20, *SD* = 4.64) than the OWNT. Nonetheless, these results were reversed in comparison to the MNT, as both metrics were higher for the U.S women. The USWNT was also losing the ball less frequently than the both the OWNT and MNT groups, a tendency illustrated by its significantly fewer miscontrols, *F*(2,202) = 18.20, *p* < .01, (*M* = 10.71, *SD* = 3.49). Furthermore, the U.S. women committed significantly fewer fouls per 90 min than the other two groups, *F*(2,202) = 51.90, *p* < .01 (*M *= 3.33, *SD *= 2.58), and lost possession less frequently than the OWNT, *F*(2,202) = 28.69, *p* < .01 (*M *= 12.71, *SD *= 3.45). Even so, men's national teams were more adept than the USWNT at retaining possession of the ball (*M *= 9.89, *SD *= 3.41).

### Differences between the NWSL and FA Women's super league

Moving into comparisons at the club level, we compared the American NWSL with its English equivalent, the FA Women's Super League (FAWSL). The results of the t-tests conducted on the groups' per-90 performance metrics are reported in Table 3. From these, we saw that NWSL teams were taking significantly more shots, *t*(360) = 19.08, *p* < .01 (*M* = 14.46, *SD* = 0.58), but the FAWSL teams were scoring significantly more goals, *t*(360) = 3.89, *p* < .01 (*M* = 1.48, *SD* = 0.58). The FAWSL group was also performing a significantly higher number of successful, *t*(360) = 7.16, *p* < .01 (*M* = 7.15, *SD* = 2.83), and unsuccessful dribbles, *t*(360) = 9.79, *p* < .01, (*M* = 10.99, *SD* = 3.31). They also passed the ball more frequently, making significantly more successful passes, *t*(360) = 24.68, *p* < .01, (*M* = 337.17.19, *SD* = 65.09), while also making fewer incomplete passes, *t*(360) = 38.12, *p* < .01 (*M* = 120.55, *SD* = 18.37).

**Table 3:** Descriptive statistics for national Women's soccer league (NWSL) and the English FA Women's super league (FAWSL) With significant comparisons.

Team	NWSL		FAWSL	
Number of games	36		326	
	Mean	SD	Mean	SD
Shots	14.46^*^	3.71	12.76	3.15
Goals	1.42	0.78	1.48^*^	0.95
Successful dribbles	5.79	2.29	7.15^*^	2.83
Unsuccessful dribbles	9.56	3.78	10.99^*^	3.31
Shots in 1st half	6.42^*^	2.42	5.94	1.93
Shots in 2nd half	8.04^*^	2.25	6.83	2.1
Goals in 1st half	0.59	0.52	0.69	0.61
Goals in 2nd half	0.82	0.67	0.78	0.63
Complete passes	306.41	34.37	337.17^*^	65.09
Incomplete passes	138.99	16.92	120.55^*^	18.37
Blocks	1.77^*^	1.02	1.17	0.67
Clearances	1.19	0.54	21.88	12.95
Interceptions	5.44	3	5.55^*^	3.2
Duels	8.14	3.39	10.19^*^	4.05
Miscontrol	14.69^*^	2.69	16.5	4.02
Dispossessions	11.49^*^	3.11	14.26	4.17
Fouls committed	9.53^*^	2.62	10.06	2.99

* *p* < .05.

Continuing, the NWSL clubs averaged significantly more blocks, *t*(360) = 6.01, *p* < .01, (*M* = 1.77, *SD* = 1.02), fewer miscontrols, *t*(360) = 17.47, *p* < .01 (*M* = 14.69, *SD* = 2.69), and more dispossessions per 90 min than the FAWSL sides, *t*(360) = 11.69, *p* < .01, (*M* = 11.49, *SD* = 3.11). The U.S clubs also committed fewer fouls, *t*(360) = 13.31, *p* < .01, (*M* = 9.53, *SD* = 2.62), but the English teams intercepted more passes, *t*(360) = 4.37, *p* < .01, (*M* = 5.55, *SD* = 3.2) and won more 50–50 duels, *t*(360) = 6.78, *p* < .01, (*M* = 10.19, *SD* = 4.05) on a 90-min basis.

## Discussion

In our efforts to more clearly define U.S. women's soccer, we discovered some notable differences between the U.S. teams and their competitors across a variety of key performance indicators. The USWNT, for example, excelled on the international stage in such areas as shot-taking, goal-scoring, passing, dribbling, and various defensive measures. As the sport of soccer itself is technically evolving, having a stronger passing and dribbling game is key to a team's success on both ends of the field (37). The USWNT was also more adept at blocking opponents' shots, and its players displayed an element of composure by clearing the ball less frequently. Fewer miscontrols and dispossessions were further indicators of the higher technical prowess that has typified the USWNT's historical dominance. In comparison to men's teams from similar international tournaments, we observed that the USWNT averaged significantly more shots, dribbles, and goals. By focusing on technical elements instead of physiological features that are known to differ, we were able to pinpoint offensive skills as staples of high-level women's soccer.

Interestingly, at the club level, a similar dominance over the chosen performance metrics was not observed. This occurred when comparing the American NWSL to the English FA Women's Super League. While the NWSL did have slightly better defensive performance with fewer miscontrols, dispossessions, and fouls committed, they were behind their English counterparts in most of the offensive metrics, only doing better in terms of shots taken per game. Having fewer goals scored from more shots taken hurts the goal-scoring efficiency, which is one area that the NWSL could potentially focus on improving. Similarly, the FAWSL also exhibited a much better passing and dribbling game, with almost 30 more completed passes per 90 min than the NWSL teams. Looking at dispossessions from a defensive standpoint, FAWSL teams might also be pressing more effectively than NWSL teams.

### Data visualization comparisons

#### Differences in shot location

**Figure 1:**
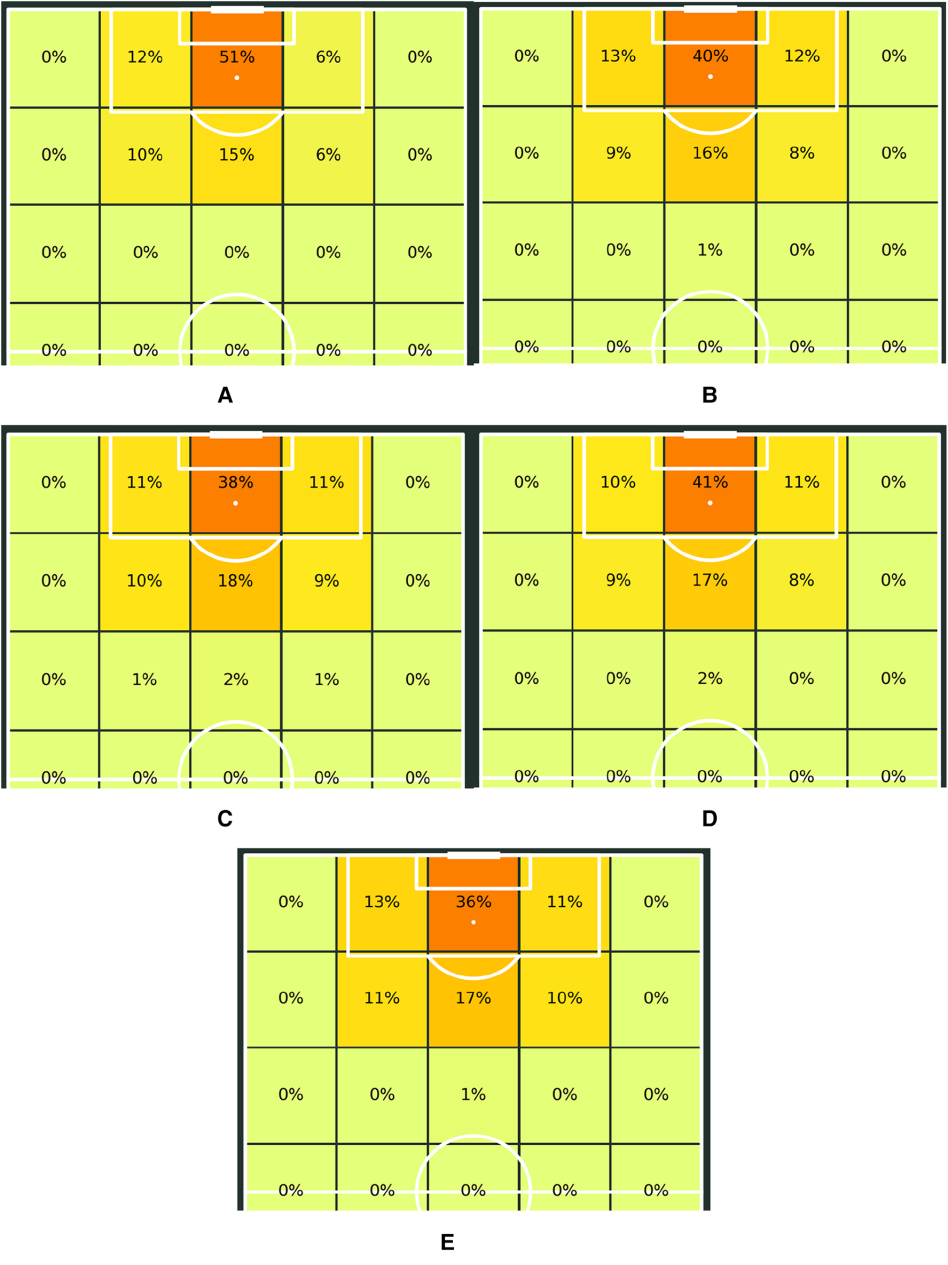
Shot frequency maps for (**A**) U.S. Women's National Team (FIFA Women's World Cup 2019), (**B**) Charlotte Courage (NWSL 2018 Winners), (**C**) Arsenal WFC (2018/19 FA WSL Winners), (**D**) The Netherlands Women's National Team (FIFA Women's World Cup 2019 Finalists), (**E**) France (FIFA Men's World Cup 2018 Winners).

Throughout the various hypotheses that were tested, some key areas that repeatedly produced significant differences were shot taking, passing, miscontrolled balls, and dispossessions. This prompted us to explore these performance metrics even further by looking at some advanced metrics and visualizations that could better explain the differences between these various groups. Regarding shot-taking, a 2-dimensional binning process was employed using the R programming language to group shots into various bins or areas on the pitch, based on the location of the shot. These shot bins were then plotted onto a pitch map to highlight the percentage of shots taken from each area. Figure 1 displays these plots.

Comparing the shot charts across the five groups, we notice that the USWNT shot the most from the region central to the goal and within the 12-yard range. Prior soccer literature has shown that this region is the one that contributes most to goal scoring (32), and the USWNT shot from this area almost 10% more frequently than any other group. The men's national teams tend to shoot the least from this region, which could be attributable to differences in the styles of play leading up to the shots, more compact defenses, or poorer decision making. The USWNT also seems to keep its shots central to the goal, whereas the NWSL seemed to favor shots from narrower angles on either side of the goal. The FAWSL, on the other hand, tended to shoot from locations that were farther away from the goal. Therefore, by analyzing the shot locations, we observe some clear distinction between the USWNT and the other women's teams and the men's national teams, which could contribute to the higher success rate that the American women traditionally get in front of the goal.

#### Differences in shot quality (expected goals)

**Figure 2:**
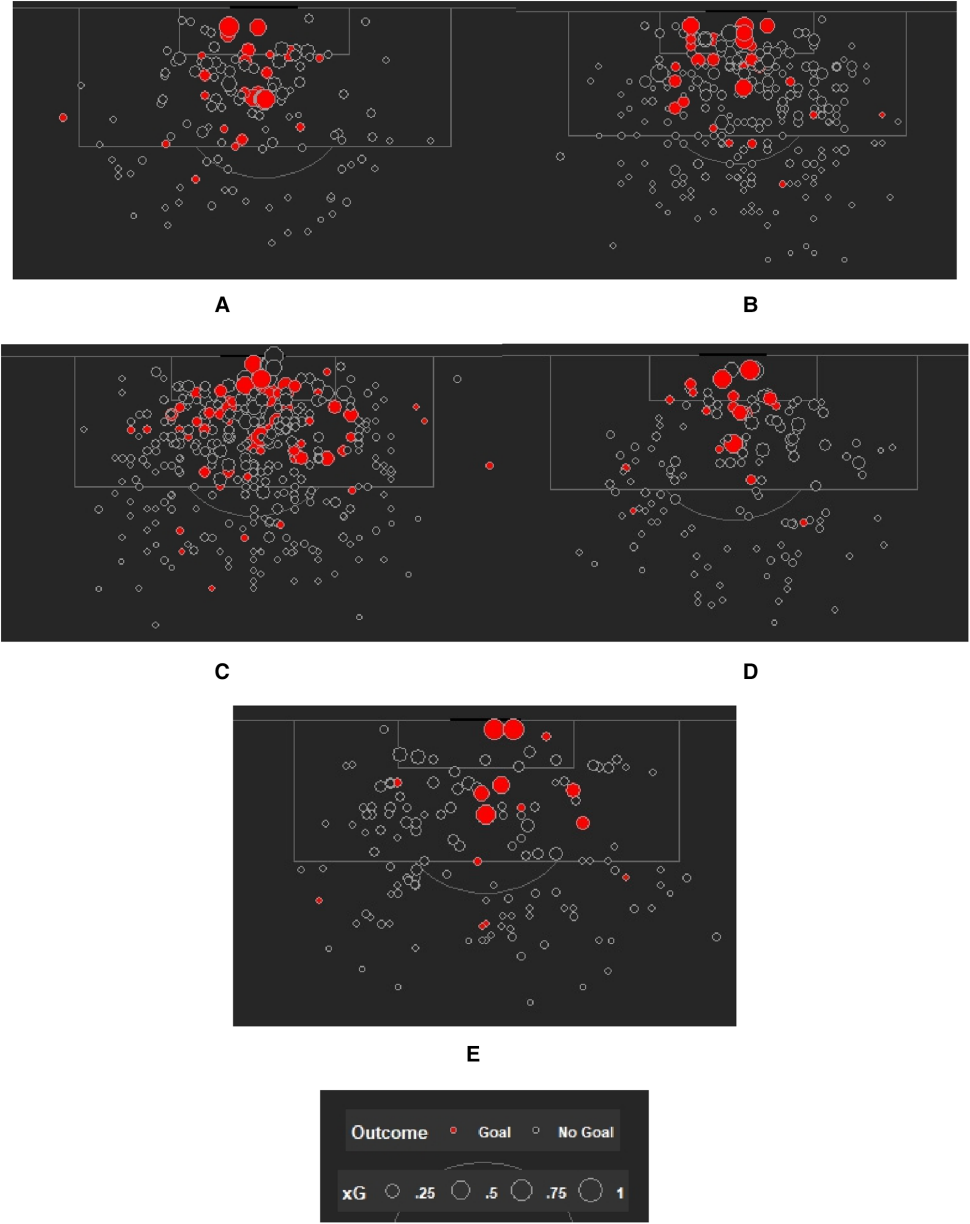
Xg shot maps for (**A**) U.S. Women's National Team (FIFA Women's World Cup 2019), (**B**) Charlotte Courage (NWSL 2018 Winners), (**C**) Arsenal WFC (2018/19 FA WSL Winners), (**D**) The Netherlands Women's National Team (FIFA Women's World Cup 2019 Finalists), (**E**) France (FIFA Men's World Cup 2018 Winners).

In a sport like soccer where the outcome is ultimately decided by an infrequent measure like goals, more advanced metrics that go deeper into the nature and quality of shot opportunities are helpful. One such metric is expected goals (xG), a stat that assigns each shot with a scoring probability based on the features (e.g., defender proximity, angle and distance to the goal, and type of preceding pass) surrounding it (38, 39). To further analyze differences across our groups, we decided to investigate what kind of shots were coming out of those regions depicted in Figure 1. Utilizing xG values assigned by StatsBomb to each shot, we plotted xG as a third variable and created various shot maps for each group. Since one of the groups only had one team (USWNT), we analyzed the shots of just one team from the other tournaments to maintain consistency. The following teams were chosen to represent each group: USWNT (2019 FIFA Women's World Cup winners); Arsenal WFC (2018/19 FAWSL winners); North Carolina Courage (2018 NWSL winners); Netherlands Women's National Team (2019 FIFA Women's World Cup finalists); France (2018 FIFA Men's World Cup winners). Figure 2 contains all the xG shot maps for the five groups.

The xG plots revealed trends that validated the initial findings observed in the shot plots. The USWNT had most of their high-xG chances from the central regions close to the goal, whereas the French national team that represented the men had shots from farther away and in wider areas to the goal. Similarly, we observed that the Arsenal WFC players were shooting from much wider/narrower angles compared to their American league counterparts on the Courage.

#### Differences in passing patterns

**Figure 3:**
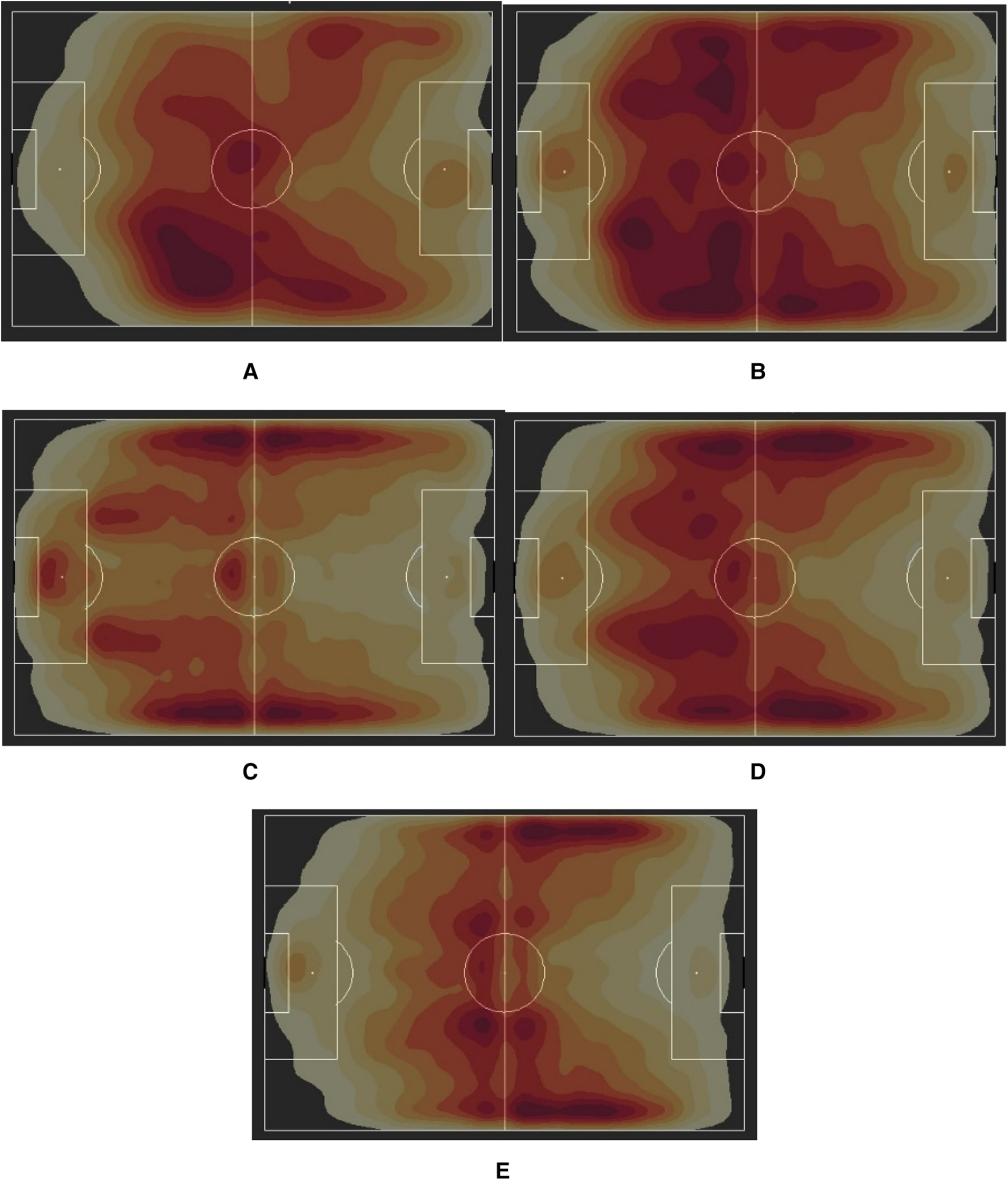
Passing heatmaps for (**A**) U.S. Women's National Team (FIFA Women's World Cup 2019), (**B**) Charlotte Courage (NWSL 2018 Winners), (**C**) Arsenal WFC (2018/19 FA WSL Winners), (**D**) The Netherlands Women's National Team (FIFA Women's World Cup 2019 Finalists), (**E**) France (FIFA Men's World Cup 2018 Winners).

Another key area of emphasis that was observed to be different across the groups was the level of passing and the efficiency of completing passes. Taking these elements into consideration, heatmaps were constructed in Figure 3 using density plots of successful pass receiving actions to see the areas on the pitch where players were more active in collecting passes. This gave us a better understanding of the respective flows of the games across these settings. From the passing heatmaps, some clear trends were established on what type of gameplay was more prevalent in each group. The USWNT, for example, seemed to rely more heavily on their right flank, engaging in a considerable amount of passing down that side. They gradually seemed to spread the play out in the final third, engaging both flanks to bring in crosses to a more central area. On the other hand, the NWSL teams seemed to take a more “play-from-the-back” approach, something that is often seen in possession-based sides like Manchester City (M) and Barcelona (M). While they also used their wide positions, the movement was much more distributed to the central areas of the pitch, emphasizing the importance of their center backs in initiating common phases of play. Other women's national teams seemed to engage in a healthy mix of flank and central play, passing from the center to the flank without as many switches as the NWSL.

Clubs in the English FAWSL stood in stark contrast to their NWSL counterparts, incorporating an approach that is almost entirely dependent on the fullbacks for ball movement. The FAWSL teams seem to rely on highly active wingers and wingbacks to create passing lanes and triangles near both sidelines before crossing it in. A similar trend was observed in the men's national teams, but one clear distinction was in how high their initial passing line was. Men's defenders seemed to push much higher up the pitch compared to the women's groups, providing better ball control for the possessing team in a crowded midfield but presenting opportunities to the opposition for swift counterattacks.

#### Differences in defensive pressures

**Figure 4:**
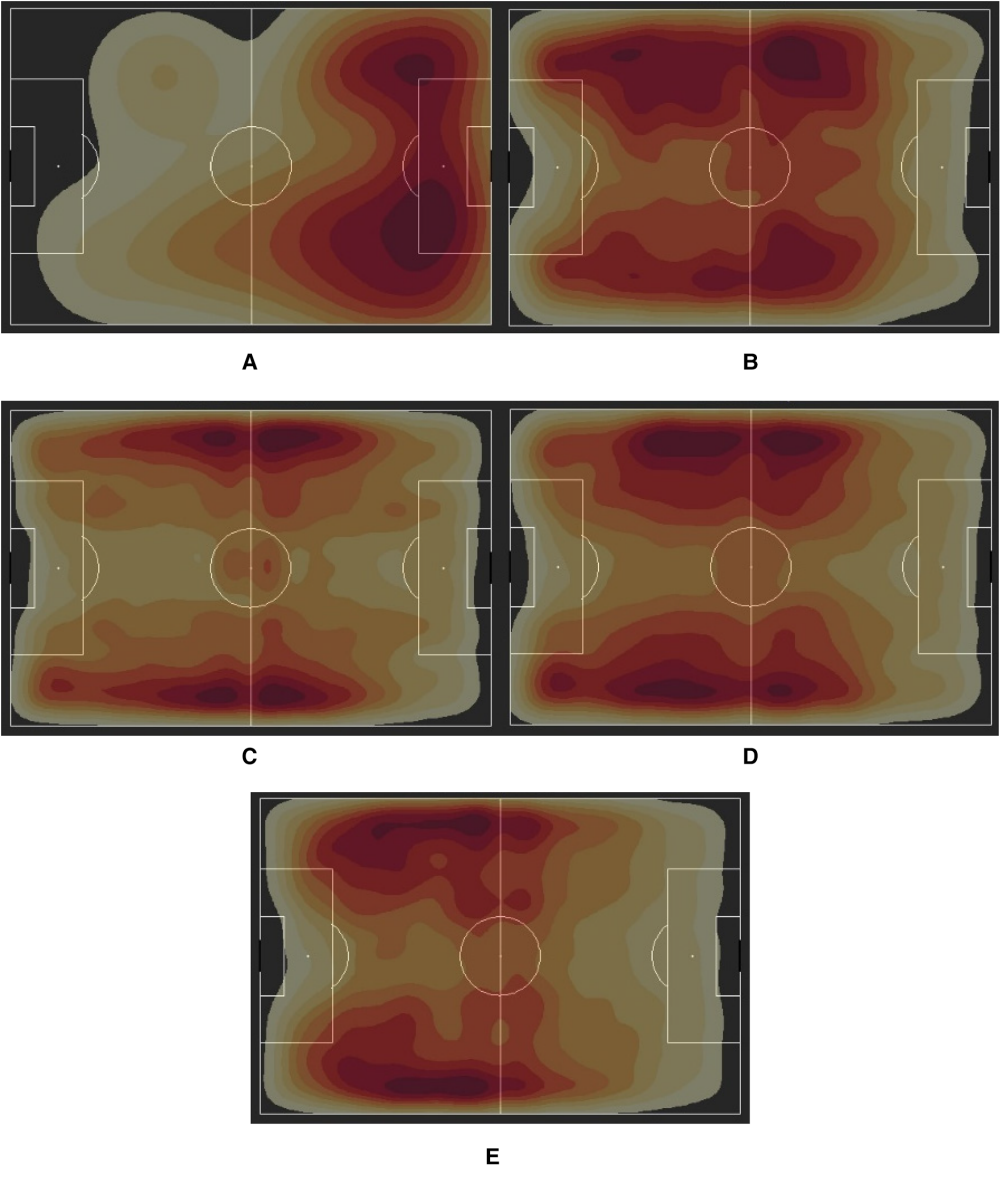
Defensive pressure heatmaps for (**A**) U.S. Women's National Team (FIFA Women's World Cup 2019), (**B**) Charlotte Courage (NWSL 2018 Winners), (**C**) Arsenal WFC (2018/19 FA WSL Winners), (**D**) The Netherlands Women's National Team (FIFA Women's World Cup 2019 Finalists), (**E**) France (FIFA Men's World Cup 2018 Winners).

Other stats that exhibited notable differences across groups were the defensive actions of blocks, interceptions, dispossessions, and miscontrolled balls. Defensive pressure, another metric captured by StatsBomb, encompasses many of these defensive elements. Without one player or many players applying pressure on the ball carrier, it is unlikely that they would miscontrol the ball or squander possession to the opposition. Therefore, similar to the pass reception heatmaps, we created defensive pressure heatmaps in Figure 4 to see where different teams were applying the most pressure. This tells us how intense and aggressive teams were in their defensive efforts.

From these, we observed that almost all the groups tended to press more on the flanks. This correlates with the increased passing observed in the previous section as more possession presents more opportunities to press and dispossess the opponent. Some key differences observed were between the NWSL and the FAWSL teams, where the NWSL pressing was much more spread out between the central areas and the flanks. Conversely, the FAWSL mainly concentrated its pressing to the wide areas. The men's national teams and most of the other women's national teams exhibited similar patterns in applying defensive pressure, sitting deeper before pressing. This suggests an emphasis on regaining defensive shape before attempting to apply pressure on the opponent.

Arguably, the most interesting case was that of the USWNT. The reigning women's World Cup champions showcased high-level intensity in defensive pressing by attacking their opponents further up the pitch. Such intense pressing prevents the other team from having the ball for more than a few seconds, resulting in unstable passing and a higher chance of opponents turning the ball over. This style of pressing is growing more popular in the modern era with managers at both the club and national level trying to adopt such intensive styles. The trade-offs in this case would be the possibility of long balls that bypass the press and physical difficulties in applying this pressure for sustained periods of the game or competition.

### Implications

Prior literature has highlighted the existence of masculine mental schemas in women's sports that detract from the characteristics that make them unique. The findings of this study move away from such male-centric orientations by seeing which technical traits are more or less prominent in U.S. women's soccer. Rigorous hypothesis testing and in-depth data visualizations across multiple offensive and defensive performance metrics revealed the characteristics of shot-taking, chance creation, dribbling, and defensive pressure that have helped establish the USWNT on an international stage. This study also elaborated on the performance levels of the NWSL and FAWSL by comparing two of women's soccer's marquee club competitions. From a broad sporting context, despite having a stellar national program in the USWNT and a head start over many of its English competitors, the NWSL still has room for growth and development in certain areas. Shooting, passing, and dribbling efficiency were not as pronounced in the NWSL. These findings stress how England and other European nations are starting to catch up to U.S. women's soccer with increased investment at the grassroots and professional levels (6). Many of Europe's established clubs have begun pouring resources into their women's teams, and recent, record-breaking attendance figures are a testament to their growth (1).

According to Metrica Sports CEO Ruben Saavedra, “The companies in the market go where the money is—and the money is in broadcasting” (40). Traditionally, the media have remained one of the biggest gatekeepers in professional sport when it comes to the content delivered to spectators. This study's results highlight offensive and defensive aspects of the USWNT's performance that should be emphasized more prominently during game broadcasts and other forms of public media. This should also guide pundits and presenters in providing a more accurate analysis of the women's game instead of using predisposed notions based on the men's equivalent. A better representation of the game by the media may ultimately translate into heightened interest among current and new fans. The study also provides a foundation for managers and analysts to take a more specific approach when working with women's soccer athletes, allowing them to address tactical strengths and weaknesses applicable to the women's game. For example, members of the USWNT have shown exceptional skill in defensive pressing, meaning they can be used as a tactical model for female personnel to try and emulate. Rather than trying to bring in strategic ideas from men's soccer, clubs such as those in the NWSL could base their tactics on schemes that have proven successful in the women's game. In doing so, women's managerial positions might be treated as platforms for growing and differentiating the sport rather than transient positions for landing similar roles in men's soccer.

### Limitations and recommendations for future research

While the results of this study have helped highlight some key performance metrics and properly frame U.S. women's soccer in comparison to its rivals, there are some areas in which future research could improve. For starters, the StatsBomb data are limited to a relatively small number of clubs, competitions, and seasons. The sample of USWNT data, for instance, only consisted of games from the 2019 World Cup. A larger, more diverse sample would increase the reliability and generalizability of the data. It would also decrease the variance in the data and help remove outliers that can skew the results. The innate differences in international play and club play should also receive further attention, and greater focus should be placed on distinct subsets of teams rather than all teams competing in a given league.

Lastly, issues related to media representation shrouding the actual performances of female athletes have come up in other sports that have both male and female athletes, such as basketball (5). While this study examines soccer, a similar analysis could be conducted in basketball to properly distinguish and frame the WNBA. Ultimately, the results of this study serve as a preliminary step into the realm of women's soccer analysis, one that could be extended by improving the sample size and looking more closely at advanced performance metrics like xG that further delineate the women's game. Deeper looks at the effects of decision making and tactical adjustments across gender could help further decode the elements that make U.S. women's soccer unique.

## Data Availability

Publicly available datasets were analyzed in this study. This data can be found here: https://github.com/statsbomb/open-data.
